# P02 Evaluating the impact of antimicrobial stewardship interventions in a UK paediatric emergency department

**DOI:** 10.1093/jacamr/dlae136.006

**Published:** 2024-08-23

**Authors:** Jathusa Peethamparam, David James, Sanjay Patel

**Affiliations:** Maidstone and Tunbridge Wells Hospital, Tunbridge Wells, UK; Paediatric Infectious Diseases, Southampton’s Children’s Hospital, University Hospital Southampton, Southampton, UK; Paediatric Emergency Medicine, Southampton’s Children’s Hospital, University Hospital Southampton, Southampton, UK

## Abstract

**Background:**

Antimicrobial resistance (AMR) poses a major threat to human health. Although rates of AMR have risen significantly in adults over the past few years, increasing rates of AMR are now being seen in children. Antimicrobial stewardship (AMS) is a highly effective approach to tackling AMR; however few paediatric AMS initiatives have focused on antibiotic prescribing in an Emergency Department (ED) setting. There are data to suggest that rates of antibiotic prescribing for respiratory tract infections (RTIs) are higher in children presenting to emergency departments than in those presenting with comparable disease severity to primary care.

**Objectives:**

The focus of this study was to evaluate the impact of two sequential antimicrobial stewardship interventions on antibiotic prescribing for children aged <16 years presenting with upper and lower respiratory tract infections to the Paediatric Emergency Department (PED) at Southampton Children’s Hospital.

**Methods:**

Antibiotic prescribing data were collected over a 16 week period (3 August 2020–22 November 2020). All children with a discharge diagnosis of upper respiratory tract infection (URTI), otitis media, tonsillitis, pneumonia and lower respiratory tract infection (LRTI) were included. Baseline data were collected between weeks 1–7. The first AMS intervention was a 15 min educational session delivered either face to face or virtually to all PED clinicians by a PED consultant (D.J.). The educational intervention used informational slide sets to reinforce the principles of judicious antibiotic use and appropriate antibiotic guideline adherence for RTIs through case-based learning scenarios and quizzes that facilitated group discussion. This intervention was repeated weekly between weeks 8–10, to ensure that all PEM staff were exposed to the intervention. The second intervention, implemented in Week 14, involved feedback of personalized antibiotic prescribing data, along with average antibiotic prescribing rates for the department, to each member of PED staff.

**Results:**

A total of 502 children with RTIs presented during the study period. Antibiotic prescribing rates significantly decreased from 28.6% during the pre-intervention period to 20.5% at the end of the study (*P*<0.05). Antibiotic prescribing for a discharge diagnosis of URTI decreased from 9.3% to 4.8% (*P*=0.11), for otitis media from 78.9% to 53.8% (*P*=0.13), for tonsillitis from 71.8% to 48.8% (*P*=0.03) and for LRTI and pneumonia from 66.7% to 51.7% (*P*=0.31).

**Conclusions:**

The combination of an educational intervention and individualized feedback of prescribing data was associated with a significant reduction in overall antibiotic prescribing for children with RTIs managed in an ED setting. However, although reductions were seen for individual pathologies, statistical significance was not always reached. This may be due to the relatively small sample size; far fewer children were recruited during the 16 week study period than predicted due to the impact of the COVID pandemic on rates of PED presentations. In general the interventions were easy to implement; however manual interrogation of patient notes was required to collect individual clinician prescribing data, this was labour intensive and would ideally be automated by electronic prescribing systems. Further work is required to show if the findings from this study can be replicated in other settings and if this impact is sustained or requires repeated AMS interventions.

